# Intelligence

**Published:** 1810-08

**Authors:** 


### INTELLIGENCE.

The Editors have the pleasure to insert the further proceedings of the Lincolnshire Benevolent Medical Society, on reformation in medical education and practice. As the Bill, it is understood, will certainly be introduced into Parliament early next winter, they are very desirous to recommend the subject to the attentive consideration of their readers, and for that purpose the Medical and Physical Journal will be open to the temperate observations of the Faculty, whether they be favourable or adverse to the plan.

" *Bull Inn, Horncastle, July* 19, 1810.

" At the Anniversary of the Lincolnshire Benevolent Medical Society, held this day, SAMUEL PITTINER, Esq. President, in the Chair: Dr. HARRISON having laid before the Meeting a printed 'Sketch of the proposed Bill for the Improvement of the Medical and Surgical, and Veterinary Sciences, and for regulating the Practice thereof,' and having given an account of the powerful encouragement and support which the Plan received during his two last visits to London, from many persons of great professional and political consideration:

" *Resolved, unanimously,*

" 1st. That this meeting is much pleased to find, that in consequence of inquiries first instituted in Lincolnshire, and subsequently extended throughout the United Kingdom, it is now generally admitted, that legislative interference is become indispensible to establish the proper distinction between well educated practitioners and others, who have assumed their titles, and filled their stations, to the great injury of society and the regular faculty.

2dly. This meeting is of opinion, that it is desirable to procure better information of the present state of medical practice, being convinced that such information, if complete, would establish beyond all controversy, the great and increasing necessity for some proof of professional competence being required from all future candidates for medical employment.

3dly. This Meeting having carefully examined the printed sketch of the Bill,* and compared it with the manuscript copy submitted to them last year, are confirmed in the unanimous sentiment then expressed—that the Bill by establishing a National Register of accredited Practitioners, by providing for the careful admission of future members of the profession; and by increasing the opportu-

* The Bill, divested of legal form, to bring it within the rules of Parliament, has been printed, and is published, together with Dr. Harrison's " Address to the Lincolnshire Benevolent Medical Society, &c."

nities for medical instruction, would, if carried into a law, become the foundation of a gradual and effectual improvement in medical science and practice.

4thly. "That the Thanks of this Meeting be given to Dr. Harrison for his unwearied exertions in the business, and that he be requested to take such further steps as appear best adapted to carry into effect this desirable object."

---

The following report was read at the last annual meeting of the Governors of the London VACCINE INSTITUTION.

"The Board of Managers have the happiness to inform the Annual Meeting, that the cause of Vaccination still continues to flourish under their auspices. This is evinced by the numerous applications for matter, not only in the Metropolis, but from all parts of the United Kingdom, the colonies, and countries abroad.

"They have great pleasure in observing the increasing progress of vaccination; their returns being greater than on any former year. It appears that since the last annual report, there have been inoculated by Dr. Walker 2,087; from the beginning 6,105. By the appointed inoculators in the metropolis, last year 1,105; from the beginning 2,163. By the appointed inoculators in the country, last year 54,665; from the beginning 156,573.

"Dr. Walker, since the last report, has supplied to 3,973 applicants, 19,865 charges of matter. From the beginning, to 12,361 applicants, 61,088 charges.

"In having been enabled to effect the foregoing incalculable services to society at large, the board of managers have to acknowledge a liberal support from a generous public; yet they owe to that public, and to the cause of vaccination, the statement of the fact, that the support of this most extensively useful establishment requires still further contributions, to enable the managers to completely effect the vast patriotic and philanthropic plan.

"It is to be lamented, that the fatal malady which has, during the last thousand years, committed ravages the most dreadful in every quarter of the world, is not yet extinct in the metropolis. The board of managers, however, request the attention of every member of the institution to this most important and gratifying circumstance; namely, that while 1163 have died of the small-pox, during the last year, as appears from the bills of mortality, not a single instance of the kind is represented as having occured from vaccination."

At a late meeting of the Royal Society, the translation of a paper by M. DELILLE was read, describing the real nature and properties of the celebrated Bohan Upas, or poison tree of Java. The author, a French physician, and a member of the National Institute of Egypt, transmitted this paper from the East Indies to the Royal Society, by an English lady. The botanical account of the plant in question he received from one of the French naturalists who accompanied Captain Baudin, and who resided some time in Java, where he visited the interior of the country, and with much difficulty difficulty prevailed on the natives to show him the different poison plants, which they carefully conceal, for the purpose of using them in war. Hence the many fabulous accounts that have been circulated respecting the fatal influence of the *Upas*; which in the language of the Javanese, signifies vegetable poison, and is applied only to the juice of the *Bohan* tree, and another plant with a twisted stem. The former is a large tree, which the writer considers as a new genus; the latter, yielding an equally powerful poison, is of the woodbine family. The *Upas*, or juice, is extracted by an incision made in the bark with a knife, and being carefully collected, is preserved by the natives to be employed in their wars. As to its diffusing noxious effluvia in the atmosphere, and destroying vegetation to a considerable distance around it, the absurdity of these stories is sufficiently exposed by the fact that the climbing species requires the support of other plants to attain its usual growth. Dr, Delille made several experiments with the upas on dogs and cats. An incision was made in the thigh of a dog, into which were dropped eight grains of the juice. The dog soon began to vomit, and continued vomiting at intervals till he became convulsed, and died in twenty minutes. Six grains were put into the thigh of another, which was seized with the same symptoms, and died in fifteen minutes. A cat was treated in like manner, but the effects were more speedy and powerful; she expired in a few minutes. All these animals died howling and in great agony. The author also made several experiments on the effects of this poison when applied internally. A grain and a half being introduced into the stomach of a dog, produced only a slight purging. To another were given four grains, which in about four hours produced the same effect, together with vomiting, and the dog died in the course of half a day. On examining the bodies of these animals after death, no very extraordinary appearances were discovered; the ventricles of the heart were full of blood, and some slight traces of inflammation appeared in the stomach; but the derangement was not so great as might have been expected from such a violent and sudden death. From this circumstance the author concluded that the absorbents had transmitted the poison to the nerves of the stomach, and that this peculiar species of vegetable poison acts exclusively on the nerves.

The MEDICAL SOCIETY of LONDON have in the press a volume of Memoirs, containing several valuable Communications in Medical and Surgical Science from eminent resident and corresponding members of the Society. The title of the volume will be, "Transactions of the Medical Society of London, Vol. I. Part I." and it will be accompanied by engravings. Part II. will appear in a few months afterwards, the Society having come to the determination of giving publicity to their Transactions more frequently than heretofore.

A new edition, being the fourth, of Dr. TROTTER's Essay, Medical, Philosophical and Chemical, on Drunkenness, and its Effects on the Human Body, with many additions, is just published.

